# A Form of Metabolic-Associated Fatty Liver Disease Associated with a Novel LIPA Variant

**DOI:** 10.34172/aim.2023.14

**Published:** 2023-02-01

**Authors:** Amir Anushiravani, Hossein Jafari Khamirani, Ashraf Mohamadkhani, Arya Mani, Mehdi Dianatpour, Reza Malekzadeh

**Affiliations:** ^1^Digestive Diseases Research Institute, Tehran University of Medical Sciences, Tehran, Iran; ^2^Department of Medical Genetics, Shiraz University of Medical Sciences, Shiraz, Iran; ^3^Cardiovascular Research Center, Department of Internal Medicine, Yale University School of Medicine, New Haven, Connecticut, USA; ^4^Department of Genetics, Yale University School of Medicine, New Haven, Connecticut, USA; ^5^Stem Cells Technology Research Center, Shiraz University of Medical Sciences, Shiraz, Iran

**Keywords:** Genetic association studies, LIPA protein, Liver cirrhosis, Lysosomal acid lipase deficiency, Non-alcoholic fatty liver disease, Whole exome sequencing

## Abstract

**Background:**

The LIPA gene on chromosome 10q23.31 contains 10 exons and encodes lipase A, the lysosomal acid lipase (LAL) containing 399 amino acids. Pathogenic variants in the LIPA result in autosomal recessive Wolman disease and cholesteryl ester storage disease (CESD). Here, we report a novel missense variant (NM_001127605.3:c.928T>A, p.Trp310Arg) of LIPA in an Iranian family with fatty liver disease identified by whole-exome sequencing and confirmed by Sanger sequencing.

**Methods:**

A 28-year-old woman referred with lean NASH cirrhosis and extremely high cholesterol levels. Fatty liver disease was found in six of her family members using vibration-controlled transient elastography (VCTE). Baseline routine laboratory tests were performed and whole-exome sequencing and confirmation by Sanger sequencing were done.

**Results:**

The index case had severe dyslipidemia and cirrhosis despite a body mass index of 21.09 kg/m^2^. Six other family members had dyslipidemia and fatty liver or cirrhosis. A homozygous missense variant (NM_001127605.3:c.928T>A, p.Trp310Arg) of LIPA which caused LAL-D was found to be associated with fatty liver disease and/or cirrhosis.

**Conclusion:**

A homozygous missense variant (NM_001127605.3:c.928T>A, p.Trp310Arg) of the LIPA gene which caused LAL-D was found to be associated with dyslipidemia, fatty liver disease and/or cirrhosis in six members of an Iranian family. These results should be confirmed by functional studies and extending the study to at least three families.

## Introduction

 During the last two decades, an epidemiologic transition has happened across the world, which is especially noticeable in developing countries, and has resulted in an obvious shift from communicable infectious diseases to chronic non-communicable diseases.^1^ One of the most common of these is fatty liver disease, previously called “non-alcoholic fatty liver disease (NAFLD)”, which has affected a quarter of the world’s population.^2^ Fatty liver disease is associated with metabolic dysfunction and obesity and it usually precedes the development of hypertension, diabetes mellitus, and cardiovascular diseases. This is why NAFLD has been redefined to metabolic-associated fatty liver disease (MAFLD).^3^

 MAFLD is a syndrome with a wide spectrum of histologic abnormalities and clinical outcomes. Although simple hepatic steatosis runs a benign clinical course, non-alcoholic steatohepatitis (NASH) may progress to cirrhosis in 25% of patients and liver-related death in 10%.^4^ Once thought to occur most commonly in obese, middle-aged Western women with diabetes, NASH is increasingly seen to affect children, normal-weight men with normal glucose levels and lipid metabolism, and persons of all ethnic groups.^5^

 The prevalence of NAFLD is increasing in Iran and the world, owing to a rising incidence of obesity and type 2 diabetes mellitus. Studies predict a 21% increase in NAFLD numbers, leading to a 33.5% overall prevalence by 2030. Coupled with a 63% increase in patients with nonalcoholic steatohepatitis (NASH), there will be a 168% increase in the number of patients with decompensated end-stage liver disease, and a 137% increase in the numbers of patients developing HCC from NAFLD. End-stage liver disease from NAFLD has become the most common reason for liver transplantation in Iran and the United States, being more prevalent than hepatitis C which has been recently treated effectively with new direct antiviral agents (DAAs).^2^

 Little is known about the heritability of hepatic ﬁbrosis, and the heritability of hepatic steatosis has not been assessed systematically in adults. Recent studies have suggested that there is a signiﬁcant genetic association with the presence of hepatic steatosis.^6-8^ The patatin-like phospholipase domain containing 3 (PNPLA-3) genotype has been linked to hepatic steatosis and also with features of NASH. However, the PNPLA-3 genotype explains 10%–12% of the variance in the trait.^9^ Therefore, 90% of the variance in the trait remains to be elucidated.

 Lysosomal acid lipase deficiency (LALD) is a rare autosomal recessive lysosomal storage disease. It is characterized by the progressive accumulation of cholesterol esters and triglycerides in the spleen and other organs.^10,11^ Few studies have suggested a strong association between impaired lysosomal acid lipase (LAL) activity and fatty liver disease.

 Here, we describe a novel mutation in the LIPA gene associated with severe fatty liver disease and cirrhosis.

## Materials and Methods

 A 28-year-old woman presented to our clinic with an asymptomatic and progressive low platelet count with elevated liver enzymes during the last two years. On physical examination, her vitals were stable. Her body mass index (BMI) was 21.09 kg/m^2^ and her spleen was palpable. She denied alcohol and herbal medicine use. Initial laboratory findings are seen in Table 1. Her family history was significant for fatty liver disease and dyslipidemia.

**Table 1:** Initial Laboratory Data of the Proband Case with Cirrhosis

**Laboratory Data**	**Value**
Platelet count	65000
AST	56 U/L
ALT	76 U/L
Total cholesterol	400 mg/dL
FPG	98 mg/dL
LDL	260 mg/dL
Alkaline phosphatase	320 U/L
Ceruloplasmin	26 mg%
Fibroscan score	27 pk
HBs Ag	Negative
HCV Ab	Negative
Immunoelectrophoresis	Normal
Upper endoscopy	Grade I esophageal varices
Abdominal sonography	Liver cirrhosis

FPG, Fasting plasma glucose; HCV Ab, hepatitis C antibody; LDL, low-density lipoprotein; AST, aspartate transaminase; ALT, alanine transaminase.

 We thereafter decided to call on all family members and screen them for a possible genetic trait. Genome-wide mapping was performed to isolate candidate loci. Seven members of this family were sequenced, all of whom were reported to have either fatty liver and/or liver cirrhosis.

 Written informed consent for physical examination and molecular investigation was obtained. Peripheral blood samples were collected from each individual for molecular and genetic tests and further investigations.

###  Exome Sequencing

 Genomic DNA was extracted from the collected samples for WES, Sanger sequencing, and further investigations. WES was carried out on the DNAs of seven members of this family. The raw data were aligned against the human reference genome (hg19) using the Burrows-Wheeler Aligner.^12^ In addition, single-nucleotide variants were called by the Genome Analysis Toolkit (GATK) software program. The variants were annotated using ANNOVAR.^13^ Some pathogenicity prediction tools were used.^14^ The patients’ phenotypes were compared to the phenotypic features associated with the candidate genes. The OMIM database was used to obtain the core phenotype associated with the identified variants (OMIM# 278000).

**Figure 1:**
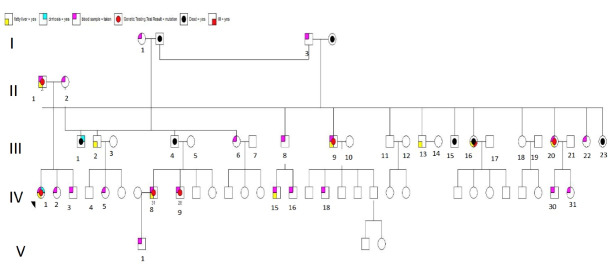
Family Pedigree.

 The family pedigree is shown in Figure 1. Clinical features are shown in Table 2. Blood and tissue samples were collected from the family members with informed consent. Routine blood tests were performed to determine the lipid proﬁle of the affected individuals, their mother, and their offspring.

**Table 2:** Family Screening and Blood Collection.

**Lab ID **	**Height (cm)**	**Weight (kg)**	**Age**	**Platelet** (**x10E3/uL)**	**FBS**	**LDL**	**HDL**	**ALT**	**AST **	**VCTE (kPa)**
III-1	167	53	28	100	71	94	43	43	42	17.5
II-1	105	75	51	254	120	147	46	86	39	5
III-9	183	84	28	264	85	140	38	40	39	7
III-8	90	183	31	211	91	134	31	85	40	5.5
II-11	172	90	60	239	123	128	31	107	70	10.5
II-22	162	66	42	198	90	140	30	46	39	
III-2	168	56	26	256	82	215	33	22	20	4
III-3	177	55	22	249	87	177	49	27	29	8
II-2	161	72	46	309	88	90	41	28	24	5
III-7										
I-1										
III-5										
II-10	160	66	64	247	88	123	47	17	20	4.5
II-24	155	60								
I-3	164	65								
III-15	170	90	31	242	89	106	45	77	63	7
III-16	185	94	35							
III-18	175	60								17.5
III-30	172	60								5
III-31	162	60								7

FBS, Fasting blood sugar; LDL, low-density lipoprotein; HDL, high-density lipoprotein; ALT, alanine transaminase; AST, aspartate aminotransferase; VCTE, vibration-controlled transient elastography.

###  Sanger Sequencing

 The Sanger sequencing results were analyzed using Codon Code Aligner. Polymerase chain reaction (PCR) was conducted to amplify the mutated sites of the genome and Oligo Primer Designer was used to design the primers. To perform PCR for the detected variant, we utilized 25 μL of the Hot start mix Ampliqon, 70 ng of DNA, 1 μL of forward primer, 1 μL of reverse primer, and 21 μL of deionized water. The DNA was amplified using the following thermocycling steps: 95°C for 15 minutes; 35 cycles of 95°C for 30 seconds, 60°C for 45 seconds and 72°C for 15 seconds; 72°C for 7 minutes.

###  Bioinformatics Analysis

**Figure 2:**
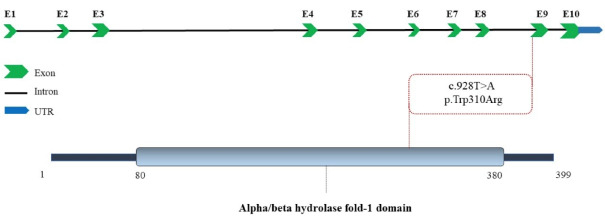
The Schematic Depiction of LIPA Gene and Lysosomal Acid Lipase/cholesteryl Ester Hydrolase Protein. The position of the novel pathogenic variant in this study (red) is shown on the gene and protein structure.

 The homozygous missense variant (NM_001127605.3:c.928T > A, p.Trp310Arg) of *LIPA* is predicted as a disease-causing or pathogenic mutation by databases like PROVEAN, SIFT, BayesDel addAF, BayesDel noAF, MetaRNN, EIGEN, EIGEN PC, FATHMM-MKL, Mutation assessor, MutPred and MVP.^14^ This variant was not listed in the gnomAD browser beta (https://gnomad.broadinstitute.org/) and ClinVar (https://www.ncbi.nlm.nih.gov/clinvar/) databases. Schematic depiction of the human *LIPA* locus with 10 exons in the top and **LAL** in the bottom. The red dash line presents the variant in present study. The two-dimensional illustration was collected from UniProt (Figure 2).

## Results

 The affected individual was a 28-year-old woman who was the product of a consanguineous marriage. The proband presented in her late 20s with weakness and fatigue and an abnormal low platelet count and elevated aminotransferases. Her vibration-controlled transient elastography (VCTE) showed fatty liver. Table 1 shows her initial laboratory data.

 Extension of the family to three generations and further clinical evaluation indicated that fatty liver was also present in one of her cousins (31 years old) and her father (51 years old) along with 2 uncles and 2 aunts. The only other family member who had cirrhosis was her uncle, without having fatty liver.

 We performed genome-wide mapping to isolate candidate loci. We sequenced 7 members of this family, all of whom were reported to have either fatty liver and/or liver cirrhosis. There were three variants that they all shared, but they are common in other populations, predicted benign by *in-silico* methods (i.e. Poly Phen2/SIFT), and/or are not well conversed across species. That being said, 6 of members of this family shared the LIPA mutation. The one who did not have this variant also had a diagnosis of fatty liver.

## Discussion

 In this study, the homozygous missense variant (NM_001127605.3:c.928T > A, p.Trp310Arg) of *LIPA *which caused LAL-D was found to be associated with fatty liver disease and/or cirrhosis. Six members of a family shared this mutation. All of them had dyslipidemia with high LDL-cholesterol levels, hepatic steatosis on their VCTE, and elevated liver enzymes, despite the fact that none were obese. One of the family members had fatty liver without the missense variant of LIPA, he was obese and had a BMI of 31.14 kg/m^2^.

 LALD is an autosomal recessive disease caused by mutations in *LIPA *at chromosomal locus 10q23.31, which encodes a hydrolase involved in the degradation of lysosomal cholesterol esters and triglycerides.^15^ The most common inherited defect is exon 8 splice junction mutation (E8SJM), which is found in more than half of patients with LALD. Jewish infants of Iraqi/Iranian origin appear to be most at risk, with an estimated incidence of 1/4200, almost making it a founder population.^16^

 Two reports in Mexico included a molecular diagnosis: a male infant affected by LALD (Mexican father and American mother) had Trp95X/ fs219 mutations; and a female infant with a sibling who died at three months had compound heterozygosity for one new mutation: p.Gln98His/p.Gly342Arg.^15^

 In the Human Mutation Database, there are only 48 *LI P A*mutations reported to date in infant (19 mutations) and child/adult (27 mutations) cases.^17^ Thus, further studies on carrier frequency of mutations in the full *LI P A*gene are required.

 LALD has been historically reported as one of two main phenotypic presentations: early onset, often called Wolman disease (WD), and late onset, termed cholesteryl ester storage disease (CESD).^18^

 Wolman disease has an estimated prevalence of 1/350,000 in infants, with symptoms such as diarrhea, massive hepatosplenomegaly, malabsorption, cachexia and adrenal calcifications, which typically develop within the first three months of life. Liver cirrhosis results in fatal liver failure before one year of age.

 The clinical signs and symptoms of CESD are heterogeneous, including hepatic steatosis leading to hepatomegaly and hepatic fibrosis and cirrhosis, splenomegaly, type Ⅱ hyperlipoproteinemia, and accelerated atherosclerosis. Although some patients remain asymptomatic until adulthood, infants and children can have significant morbidity and early mortality as a result of liver cirrhosis and liver failure.^3^ The early signs of portal hypertension and its correlation to hepatic fibrosis and the progression of disease are unknown. Although liver manifestations of the disease usually predominate, dyslipidemia and associated cardiac complications have been also reported. The principal causes of death reported in CESD patients were liver failure and bleeding of esophageal varices. However, bleeding of esophageal varices was reported in only 8% of patients, and this complication is primarily described in Mexican patients.^4^

 Another Mexican study showed that a decrease in LAL activity was associated with significant liver fibrosis and cirrhosis at levels < 0.265 nmol/spot/h.^19^

 Treatment for LALD has been limited to the use of lipid-lowering drugs or hepatic transplantation, which was performed in nine patients with mixed-results but with no long-term follow-up.^20^ Statins do help with LDL cholesterol. However, in studies that have looked at what happens to the liver, progressive liver disease is seen in these patients. Statins can be helpful in decreasing LDL cholesterol, but liver disease continues to progress. Liver transplantation has been investigated in a small series of studies. No long-term data is available. There are about 9 patients in the literature ranging from the ages of 5 to 14 years. Again, this is a multi-organ disease, so we do not believe that a liver transplant only is going to be very beneficial.^21^

 Sebelipase alfa (Synageva BioPhar ma Corp., Lexington, MA, United States), a recombinant human LAL, is under development for use as enzymatic replacement therapy. The initial trial in adults showed a decrease in serum lipids and liver volume and normalization of liver transaminases,^22^ and a phase 3 global trial is currently under way.^23^ Timely diagnosis, as well as incorporation of LAL activity in newborn screening, will be needed to optimize the time to begin enzymatic replacement therapy. Further research is needed for evaluating additional therapies for CESD patients, such as bone marrow and hepatic transplantation.

 LALD has a variable age of onset and is often under-recognized. The symptoms may include hepatomegaly, elevated liver enzymes, and dyslipidemia. Diagnosis can be made based on the LAL activity or the lipomutation analysis, and enzyme replacement therapy is currently being investigated.^20^ It is important to make the diagnosis because it has implications for liver monitoring and potentially for therapeutic approaches under development.

 Our study had limitations; the sample size was small and functional analysis on LAL-A is also needed to strengthen the results. VCTE has its limitations in estimating hepatic steatosis and fibrosis, but liver biopsy, which is the gold standard, is invasive and patients usually resist this procedure. A liver MRI may have increased sensitivity and specificity for our measures but is expensive.

 In conclusion, MAFLD is the most common liver disease worldwide and is the number one etiology of liver transplantation. Obesity and the metabolic syndrome have a major role, but there is growing evidence supporting a role for the heritability of fatty liver diseases, especially in those with lean NASH.

 We found a homozygous missense variant (NM_001127605.3:c.928T > A, p.Trp310Arg) of the *LIPA *genewhich caused LAL-D to be associated with dyslipidemia, fatty liver disease and/or cirrhosis in six members of a family. These results should be confirmed by functional studies and extending the study to at least three families.
